# Shenfu injection combined with furosemide in the treatment of chronic heart failure in patients with coronary heart disease: A protocol of randomized controlled trial

**DOI:** 10.1097/MD.0000000000024113

**Published:** 2021-01-22

**Authors:** Yibing Gao, Ying Gao, Rong Zhu, Xiao Tan

**Affiliations:** Department of Emergency Medicine, The Second Affiliated Hospital of Nanjing Medical University, Nanjing 210011, Jiangsu Province, China.

**Keywords:** chronic heart failure, combination therapy, coronary heart disease, furosemide, shenfu injection

## Abstract

**Background:**

Coronary heart disease (CHD) is an important cause of chronic heart failure, and chronic heart failure is also a serious complication in the end stage of coronary heart disease. At present, there is no specific treatment plan. Shenfu injection has advantages in the treatment of heart failure in patients with coronary heart disease, but there is a lack of standard clinical study to verify this. Therefore, the purpose of this randomized controlled trial is to evaluate the efficacy and safety of Shenfu injection combined with furosemide in the treatment of chronic heart failure in patients with coronary heart disease.

**Methods:**

This is a prospective randomized controlled trial to study the efficacy and safety of Shenfu injection combined with furosemide in the treatment of coronary heart disease and chronic heart failure. This study will be approved by the clinical research ethics committee of our hospital. The patients will be randomly divided into two groups according to 1:1:(a) Shenfu injection combined with furosemide group and (b) simple furosemide group. Standard treatment for 7 days followed up for 30 days at the same time, pay attention to its efficacy and safety indicators. The total effective rate of cardiac function improvement, left ventricular ejection fraction (LVEF), N-terminal pro-brain natriuretic peptide (NT-pro BNP), 6-minute walk test (6-MWTD), and adverse reactions will be observed. Data will be analyzed using the statistical software package SPSS version 25.0 (Chicago, IL).

**Discussion:**

This study will evaluate the efficacy and safety of Shenfu injection combined with furosemide in the treatment of coronary heart disease with chronic heart failure. The results of this experiment will provide a clinical basis for Shenfu injection combined with furosemide in the treatment of coronary heart disease and chronic heart failure.

**OSF Registration number:**

doi: 10.17605/OSF.IO/27FPM

## 1 Introdoction

Chronic heart failure is a common clinical syndrome characterized by dyspnea, fatigue, and signs of volume overload, which may include peripheral edema and pulmonary rales.^[1]^ In developed countries,about 2% of adults suffer from heart failure, and the incidence increases to more than 10% in people aged 70 years and older.^[2]^ Despite the continuous improvement of treatment, chronic heart failure remains a disease with poor prognosis. In 5 years of observation, the mortality rate was 50%.^[2]^ The most important cause of chronic heart failure is coronary heart disease.^[3]^ At the same time, chronic heart failure is one of the most serious complications of coronary heart disease.^[4]^

In order to improve the symptoms and exercise abilities in patients with chronic heart failure, the guidelines developed by the European Heart Association recommend the use of diuretics,^[2]^ which can reduce the number of readmissions associated with heart failure through appropriate fluid retention management.^[5]^ In the clinic, the most commonly used diuretic in patients with heart failure is loop diuretic furosemide (furosemide). However, some studies have pointed out that furosemide has no positive effect on the prognosis of patients, and may even be related to an increased risk of hospitalization, all-cause mortality, and cardiovascular mortality.^[6]^

In recent years, Chinese medicine has shown great advantages in the treatment of chronic heart failure.^[7]^ According to the theory of TCM, the main cause of chronic heart failure is deficiency of heart Qi caused by Qi deficiency and blood stasis. Shenfu Injection (SFI) is a kind of special traditional Chinese medicine extracted from ginseng and aconite, which has the function of invigorating Qi and promoting blood circulation. SFI has shown a variety of pharmacological activities, including increased blood pressure and enhanced myocardial contractility. It has been used in China for the treatment of cardiovascular disease patients with reliable efficacy for many years.^[8]^ At present, there are no studies on the efficacy and safety of Shenfu injection combined with furosemide in the treatment of coronary heart disease with chronic heart failure. Therefore, we intend to evaluate the efficacy and safety of Shenfu injection combined with furosemide in the treatment of coronary heart disease and chronic heart failure through this randomized controlled trial.

## 2 Materials and methods

### 2.1 Study design

**Figure 1:**
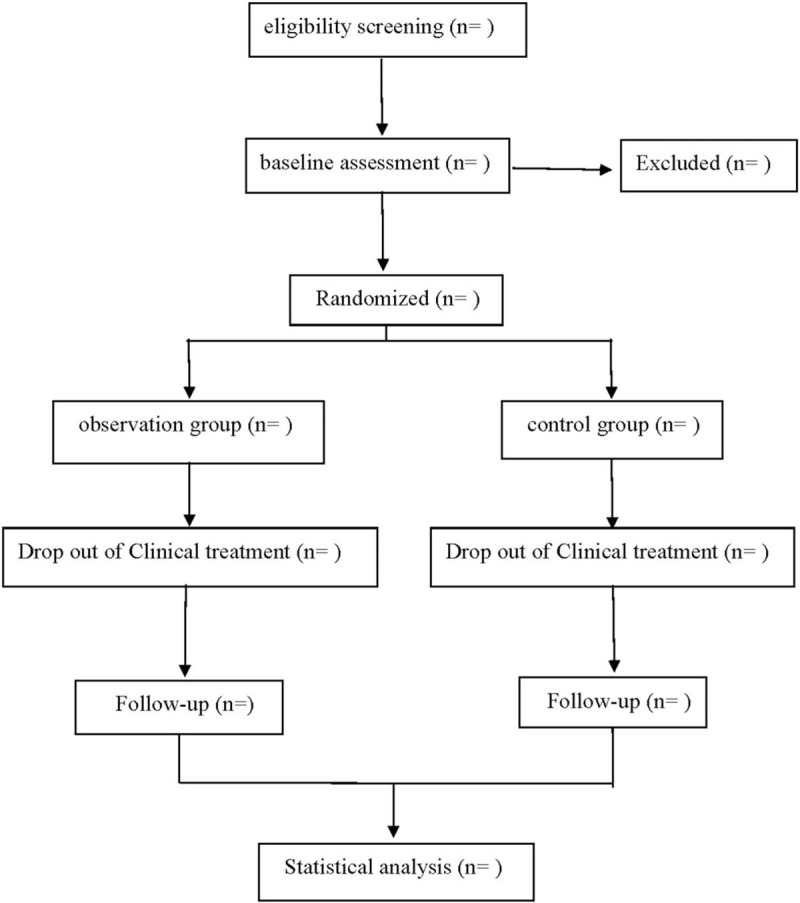
Flow diagram.

This is a prospective randomized controlled trial to study the efficacy and safety of Shenfu injection combined with furosemide in the treatment of coronary heart disease with chronic heart failure. This study will follow the Consolidated Standards of Reporting Trials (CONSORT).^[9]^ Its flow chart is shown in Figure 1.

### 2.2 Ethics and registration

The protocol will be in accordance with the Helsinki Declaration and approved by the Clinical Research Ethics Committee of our hospital. This experiment has been registered in the open science framework (OSF) (registration number: doi: 10.17605/OSF.IO/27FPM). Before randomization, all patients will sign a written informed consent. and they can freely choose whether to continue the trial at any time.

### 2.3 Patients

Inclusion criteria: ① meet the diagnostic criteria of coronary heart disease, the patients had occlusive myocardial infarction, and the stenosis rate of the main branch of the coronary artery (at least one branch) is more than 50%;) ② meet the diagnostic criteria for chronic heart failure.^[2]^ Doppler echocardiography indicated that the left ventricular ejection fraction was less than 50%;^[10]^③ The age was 40 to 79 years; ④ New York Heart Association (NYHA) grade III–IV; ⑤ No myocardial infarction and angina pectoris occurred in the past month.

Exclusion criteria: ① Patients with heart failure induced by other diseases such as congenital heart disease; ② Complicated with other serious diseases such as malignant tumor; ③ Other heart diseases such as severe arrhythmia; ④ Complicated with serious mental illness; ⑤ Patients with drug allergy or contraindications in this study; ⑥ Unable to understand the study protocol or unwilling to participate in the study after explanation.

### 2.4 Sample size calculation

This study is a pilot clinical trial. The calculation of the sample size will be based on a drop-out rate of 20% in the clinical study, and 0.025% of the sample size will be taken as 90%, which is calculated to include 110 patients. The patient will randomly select any number from 001 to 110 from the sealed envelope and is assigned to the observation group (odd) and the control group (even) according to the parity of the number.

### 2.5 Study design

In this study, patients who meet the criteria will be screened through pre-hospital recruitments and in-hospital inpatients. The patients and their families will approve the study program and sign the informed consent form. The observation group will receive SFI (SFI 50 mg / kg CS100 ml, ivgtt, qd) combined with furosemide injection (Furosemide 20 mg / kg GS 250 ml, ivgtt, qd), while the control group will only receive furosemide injection (Furosemide 20 mg / kg GS 250 ml, ivgtt, qd). Patients in both groups will receive the same routine care and drug treatment, such as angiotensin converting enzymeinhibitiors (ACEIs) or angiotensin II receptor blockers (ARBs). If necessary, the attending physician can adjust the plan according to the patient's condition, and all interventions will be recorded in detail for final result analysis. We will set up a special drug manager, and the nurse will be responsible for preparing the medicine, preparing the solution, and using the disposable optical infusion device to infuse the corresponding numbered patients. Therefore, the study is not blind to nurses, but to researchers, patients, and statisticians. All patients will receive intravenous injections once a week. The health status of each patient will be evaluated before and after treatment, including efficacy and safety indicators, and all patients will be followed up for 30 d by telephone or outpatient clinic. Follow-up will include cardiovascular events and rehospitalization.

### 2.6 Evaluation criteria and efficacy judgment

1. Main outcome measures: ① total effective rate of cardiac function improvement (refer to the guiding principles of clinical research of new drugs in traditional Chinese medicine^[11]^), Excellent: heart failure was essentially ameliorated or the NYHA classification increased by at least 2 levels; Valid: NYHA classification increased by 1 level; Invalid: NYHA classification remained the same before and after the treatment. Worsened: NYHA classification decreased by at least.
2. Secondary outcome measures: left ventricular ejection fraction (LVEF), N-terminal pro-brain natriuretic peptide (NT-pro BNP), tumor necrosis factor-*α* (TNF-*α*), Interleukin-6(IL-6), and 6-minute walk test.
3. Adverse reactions, including abnormal liver and kidney function and any discomfort during treatment (such as dizziness, nausea, etc.).

### 2.7 Data collection and management

Data will be collected before treatment, 72 hours after treatment, and at the end of treatment according to evaluation criteria. After 30 days of treatment, outpatient or telephone follow-up will be conducted for each patient. Detailed follow-up information can not be collected to record the reasons for loss of follow-up information. All data will be collected by one or two assistants. Personal information about potential and registered participants will be collected, shared, and kept in a separate storeroom to protect confidentiality before, during, and after the trial. Access to the database will be limited to the researchers of this research group.

### 2.8 Statistical analysis

Data will be analyzed according to the full analysis set principle. Fisher exact test and the Mann–Whitney U test will be performed for comparison of both groups, for categorical variables and continuous variables, respectively. When *P* < .05, the difference will be statistically significant.

## 3 Discussion

Chronic heart failure is the main cause of hospitalization in the elderly worldwide, with high mortality, and has a negative impact on the quality of life in patients.^[12]^ Patients with coronary heart disease are affected by coronary arteriosclerosis, stenosis, or obstruction for a long time, which can easily cause myocardial ischemia damage, lead to heart failure, and increase mortality. Integrated traditional Chinese and Western medicine treatments are a hot spot in modern medicine.

As a traditional Chinese medicine, Shenfu injection has long been used in the treatment of coronary heart disease and heart failure in China, and has achieved good results.^[13]^ Animal studies have shown that Shenfu injection can reduce tumor necrosis factor in the blood of rats with heart failure -*α* (TNF-*α*). The therapeutic mechanism may be related to the reduction of apoptosis and the inhibition of related factors.^[14]^ A randomized controlled study confirmed that Shenfu injection can significantly improve the clinical symptoms and myocardial tolerance of patients with congestive heart failure in the acute phase, without adverse reactions.^[8]^ Since there is no standard clinical study to evaluate the efficacy of Shenfu injection combined with diuretics in the treatment of chronic heart failure in patients with coronary heart disease, we intend to evaluate its efficacy and safety through prospective randomized controlled trials.

This study also has some limitations. Due to the short planned follow-up time, we cannot understand the impact of long-term efficacy. Therefore, we may extend the follow-up time if necessary. At the same time, due to the influence of treatment methods, this study cannot be strictly double-blind, which may have a certain impact on the results.

## Author contributions

**Data curation:** Yibing Gao, Ying Gao.

**Funding acquisition:** Xiao Tan.

**Investigation:** Ying Gao.

**Resources:** Rong Zhu, Xiao Tan.

**Software:** Rong Zhu.

**Supervision:** Yibing Gao, Rong Zhu.

**Writing – original draft:** Yibing Gao, Ying Gao.

**Writing – review & editing:** Yibing Gao, Xiao Tan.
